# Constrained Deep Q-Learning Gradually Approaching Ordinary Q-Learning

**DOI:** 10.3389/fnbot.2019.00103

**Published:** 2019-12-10

**Authors:** Shota Ohnishi, Eiji Uchibe, Yotaro Yamaguchi, Kosuke Nakanishi, Yuji Yasui, Shin Ishii

**Affiliations:** ^1^Department of Systems Science, Graduate School of Informatics, Kyoto University, Now Affiliated With Panasonic Co., Ltd., Kyoto, Japan; ^2^ATR Computational Neuroscience Laboratories, Kyoto, Japan; ^3^Department of Systems Science, Graduate School of Informatics, Kyoto University, Kyoto, Japan; ^4^Honda R&D Co., Ltd., Saitama, Japan

**Keywords:** deep reinforcement learning, deep Q network, regularization, learning stabilization, target network, constrained reinforcement learning

## Abstract

A deep Q network (DQN) (Mnih et al., 2013) is an extension of Q learning, which is a typical deep reinforcement learning method. In DQN, a Q function expresses all action values under all states, and it is approximated using a convolutional neural network. Using the approximated Q function, an optimal policy can be derived. In DQN, a target network, which calculates a target value and is updated by the Q function at regular intervals, is introduced to stabilize the learning process. A less frequent updates of the target network would result in a more stable learning process. However, because the target value is not propagated unless the target network is updated, DQN usually requires a large number of samples. In this study, we proposed Constrained DQN that uses the difference between the outputs of the Q function and the target network as a constraint on the target value. Constrained DQN updates parameters conservatively when the difference between the outputs of the Q function and the target network is large, and it updates them aggressively when this difference is small. In the proposed method, as learning progresses, the number of times that the constraints are activated decreases. Consequently, the update method gradually approaches conventional Q learning. We found that Constrained DQN converges with a smaller training dataset than in the case of DQN and that it is robust against changes in the update frequency of the target network and settings of a certain parameter of the optimizer. Although Constrained DQN alone does not show better performance in comparison to integrated approaches nor distributed methods, experimental results show that Constrained DQN can be used as an additional components to those methods.

## 1. Introduction

In recent years, considerable research has focused on deep reinforcement learning (DRL), which combines reinforcement learning and deep learning. Reinforcement learning is a framework for obtaining a behavioral policy that maximizes value through trial and error, even under unknown conditions. Deep learning is a method of high-level pattern recognition, and it has demonstrated efficient performance in a variety of image-processing problems. DRL has been applied to various optimization problems, such as robot (Levine et al., 2016) and drone control (Kahn et al., 2017) and game learning (Mnih et al., 2013). Alpha Go (Silver et al., 2016) is one of the most well-known applications of DRL. Alpha Go was created in 2016, and it later defeated Lee Sedol (9 dan, or 9th level) and Ke Jie (9 dan), the world's best Go players. The newest version of Alpha Go outplays the previous version without prior knowledge of the positions of the stones or historical records of human actions during play (Silver et al., 2017). However, use of DRL still faces several unresolved problems. These problems include the requirement for a very large number of samples, the inability to plan a long-term strategy, and the tendency to perform risky actions in actual experiments.

The original DRL is a Deep Q Network (DQN) (Mnih et al., 2013, 2015) proposed by Google Deep Mind, which learned to play 49 different Atari 2600 games simply through a game screen. Q learning (Watkins and Dayan, 1992; Sutton and Barto, 1998) is a typical reinforcement learning method. In Q learning, an optimal action policy is obtained after learning an action value function (a.k.a. Q function). DQN uses a convolutional neural network (CNN) to extract features from a screen and Q learning to learn game play. Considerable research has been conducted on expanded versions of DQN, such as double Q learning (van Hasselt, 2010; van Hasselt et al., 2016) that reduces overestimation of the action values, prioritized experience replay (Schaul et al., 2015), which gives priority to experience data used for learning, dueling network architecture (Wang et al., 2016), which outputs action values from state values and advantage values, and the asynchronous learning method with multiple agents (Mnih et al., 2016). Rainbow DDQN (Hessel et al., 2017) combines several DQN extensions: Double DQN, prioritized experience replay, dueling network, multi-step bootstrap targets, Noisy Net (Fortunato et al., 2018) that injects noise into the networks' weights for exploration, and Distributional DQN that models the distribution whose expectation is the action value. Ape-X DDQN (Horgan et al., 2018) is a distributed DQN architecture, as in which distributed actors are separated from the value leaner, and it employs Double DQN, dueling network architecture, distributed prioritized experience replay, and multi-step bootstrap targets. Recurrent Replay Distributed DQN (R2D2) is one of the state-of-the-art distributed architecture that proposes distributed prioritized experience replay when the value function is approximated by recurrent neural networks (Kapturowski et al., 2019).

When attempting to find the optimal Q function within a class of non-linear functions, such as neural networks, learning becomes unstable or in some cases does not converge (Tsitsiklis and Van Roy, 1997). In DQN, learning is stabilized through a heuristic called experience replay (Lin, 1993) and the use of a target network. Experience replay is a technique that saves time-series data in a buffer called replay memory. In experience replay, mini batch learning is performed using randomly sampled data from the buffer. Consequently, the correlations between the training samples are reduced, and thus the learning is stabilized. The target network is a neural network, which is updated with a slower cycle for the neural network representing the Q function. By using a fixed target network to calculate the target value, we can expect stabilization of the entire learning process. In general, a less frequent updates of the target network would result in a more stable learning process. For example, Hernandez-Garcia (2019) and Hernandez-Garcia and Sutton (2019) reported that decreasing the update frequency from 2,000 to 500 steadily reduced the instability of the algorithm. van Hasselt et al. (2016) increased the update frequency of the target network from 10,000 to 30,000 to reduce overestimation of the action values. It is known that using the target network technique disrupts online reinforcement learning and slows down learning because the value is not propagated unless the target network is updated (Lillicrap et al., 2016; Kim et al., 2019). Consequently, the number of samples required for learning becomes extremely large.

To stabilize the learning processes in DQN, Durugkar and Stone (2017) proposed Constrained Temporal Difference (CTD) algorithm to prevent the average target value from changing after an update by using the gradient projection technique. The CTD algorithm was validated by showing convergence on Baird's counterexample (Baird, 1995) and a grid-world navigation task, although the CTD algorithm did not require a target network. However, Pohlen et al. (2018) and Achiam et al. (2019) showed that the CTD algorithm did not work well in more complicated problems. Pohlen et al. (2018) proposed the Temporal Consistency loss (TC-loss) method, which tries to prevent the Q function at each target state-action pair from changing substantially by minimizing changes of the target network. Although the use of high discount factors usually leads to propagation of errors and instabilities, it has been shown that DQN with TC-loss can learn stably even with high discount factors.

In this study, we focus on this problem and propose a method with practically improved sample efficiency. In our proposed method, a standard TD error is adopted for bootstrapping, while the difference between the value of the best action of the learning network and that of the target network is used as a constraint for stabilizing the learning process. We call this method Constrained DQN. When the difference between the maximum value of the Q function and the corresponding value of the target network is large, Constrained DQN updates the Q function more conservatively, and when this difference is sufficiently small, Constrained DQN behaves in the manner of Q learning. As learning progresses, the number of times the constraints are activated decreases, and DQN gradually approaches conventional Q learning. Using this method, we expect an acceleration of convergence in the early stage of learning by reducing the delay in updating the Q function, since the target value is calculated without using the target network. In addition, we expect the results to be equivalent to those of Q learning without constraints when learning is completed. We applied our Constrained DQN to several tasks, such as some Atari games and a couple of control problems with a discrete state space and a continuous state space, respectively. Consequently, Constrained DQN converged with fewer samples than did DQN, and it was robust against fluctuations in the frequency of updates in the target network. Although Constrained DQN alone does not show better performance in comparison to integrated approaches, such as Rainbow nor distributed methods like R2D2, experimental results show that Constrained DQN can be used as an additional component to those methods.

## 2. Background

In this section, we give an outline of Q learning, which is a typical method of value-based reinforcement learning and forms the basis of the proposed method. Variants of Q learning are also discussed here.

### 2.1. Reinforcement Learning

The agent is placed in an unknown environment, observes a state of the environment at each time step, and receives a reward following the selected action. Mapping a state to an action is called a policy. The objective of reinforcement learning is to determine the optimal policy that allows the agent to choose an action that maximizes the expected sum of rewards received in the future. The state of the environment is represented as *s*, and the agent probabilistically selects an action *a* based on stochastic policy π(*a* ∣ *s*) = Pr(*a* ∣ *s*). After the action selection, the state changes to *s*′ according to the transition probability Pr(*s*′ ∣ *s, a*), and thus a reward *r*(*s, a, s*′) is obtained.

When the state transition has a Markov property, this problem setting is called a Markov Decision Process (MDP). On the other hand, the environmental state *s* may not be observed directly or may only be partially observed. In such a case, the agent must predict state *s* from observation *o*. When the mapping of *s* to *o* is stochastic, this problem setting is called a Partially Observable Markov Decision Process (POMDP). As a solution to POMDP, we use the history of past observations *o* as a pseudo state and then apply a reinforcement learning method as if the pseudo state constituted an ordinary MDP. The Atari game, to which DQN was first applied, is a POMDP problem because the current game screen alone cannot uniquely represent the game state. However, in Mnih et al. (2015), the most recent set of four consecutive observations was used as a pseudo state to approximately handle the Atari game play as an MDP.

### 2.2. Q Learning With Function Approximation

To illustrate the difference between Q learning and DQN, we briefly explain the basic algorithm here. We define the sum of discounted rewards obtained after time *t* as the return *R*_*t*_ as follows:

$$\begin{array}{l} R_{t}=\overset{\infty }{\underset{k=0}{\sum }}\gamma^{k}r_{t+k}, \end{array}$$

where 0 ≤ γ < 1 is the discount rate for future rewards. The smaller the value of γ, the more emphasis is placed on the reward in the near future. The action value function (Q function) is then defined by

$$\begin{array}{l} Q^{\pi }\left(s,a\right)=E_{\pi }\left\{R_{t}|s_{t}=s,a_{t}=a\right\}, \end{array}$$

where *E*_π_{…} represents the expectation under stochastic policy π. The Q function *Q*^π^(*s, a*) represents the expected sum of discounted rewards when the agent chooses action *a* under state *s* and then selects actions according to policy π. The Q function is described as the following recursive formula:

$$\begin{array}{l} Q^{\pi }\left(s,a\right)=\underset{s^{\prime }\in S}{\sum }\mathrm{Pr}\left(s^{\prime }|s,a\right)\left(r\left(s,a,s^{\prime }\right)+\gamma \underset{a^{\prime }\in A}{\sum }\pi \left(a^{\prime }|s^{\prime }\right)Q^{\pi }\left(s^{\prime },a^{\prime }\right)\right), \end{array}$$

where *S* and *A* are the state set and the action set, respectively. From this formula, we can determine that the Q function under the optimal policy π^*^, i.e., the optimal Q function, satisfies the following equation, which is known as the Bellman optimality equation:

(1) $$\begin{array}{r} Q^{*}\left(s,a\right)=E_{s^{\prime }}\left\{r_{t}+\gamma \underset{a^{\prime }}{\;\max}Q^{*}\left(s^{\prime },a^{\prime }\right)\right\}. \end{array}$$

In Q learning, by iteratively updating the Q function using (1) based on empirical data, the Q function can be stochastically converged to Q^*^(*s, a*), and so the optimal policy can be determined as the policy that is greedy with respect to Q^*^: $a^{*}={\text{argmax}}_{a}Q^{*}\left(s,a\right)$. In practice, a learning agent has to explore the environment because the Q function is not reliable, and ϵ-greedy action selection has been widely used as a stochastic policy to probabilistically select an action *a* for an input state *s*. More specifically, the ϵ greedy policy selects an action that maximizes the Q function at state *s* with a probability of 1 − ϵ, ϵ ∈ [0, 1] and chooses a random action with the remaining probability. ϵ was initially set to 1.0 and gradually reduced as learning progressed, and it was fixed after becoming a small value like 0.1. Consequently, at the beginning of learning, various actions were searched at random, and as learning progressed, good actions were selectively performed based on the action value function that had become more reliable.

When the states and actions are discrete and finite, a simple way to represent the Q function is to use a table of values for all pairs of states and actions. The table is arbitrarily initialized and updated with data on the agent experience as follows:

$$\begin{array}{l} Q\left(s,a\right)\leftarrow Q\left(s,a\right)+\alpha \left(r+\gamma \underset{a^{\prime }}{\;\max}Q\left(s^{\prime },a^{\prime }\right)-Q\left(s,a\right)\right), \end{array}$$

where 0 < α ≤ 1 is the learning rate, and the larger the learning rate, the stronger is the influence of new data for updating. With this learning algorithm, the Q table converges to the optimal Q function under the convergence condition of stochastic approximation. On the other hand, because this is based on the stochastic approximation method, a sufficient number of data for all pairs of (*s, a*) is required.

In tabular Q learning, when the number of elements in the state or action space is enormous or the state or action space is continuous, we often express the Q function as a parametric function *Q*(*s, a*; θ) using the parameters θ and then update the parameters according to the gradient method:

(2) $$\begin{array}{r} \theta \leftarrow \theta +\alpha \left(\text{targe}{\text{t}}_{Q}-Q\left(s,a;\theta \right)\right)\nabla_{\theta }Q\left(s,a;\theta \right). \end{array}$$

Here, “target_*Q*_” is a target value based on the optimal Bellman Equation (1), and it is calculated as follows:

$$\begin{array}{r} \text{targe}{\text{t}}_{Q}=r\left(s,a,s^{\prime }\right)+\gamma \underset{a^{\prime }}{\;\max}Q\left(s^{\prime },a^{\prime };\theta \right). \end{array}$$

The Q function is updated according to its self-consistent equation. Q-learning is a bootstrap method, where the Q function approximator is regressed toward the target value, which depends on itself. This implies that the target value changes automatically when the learning network is updated. Therefore, when a non-linear function, such as a neural network is used for approximation, this learning process becomes unstable due to dynamical changes in the target, and in the worst case, the Q function diverges (Sutton and Barto, 1998).

## 3. Extension of Q Learning

### 3.1. Deep Q Network

To prevent the Q function from diverging, DQN introduces a separate target network that is a copy of the Q function, and this is used to calculate the target value. In this case, the Q function is updated as follows:

$$\begin{array}{l} \theta \leftarrow \theta +\alpha \left(\text{targe}{\text{t}}_{\text{DQN}}-Q\left(s,a;\theta \right)\right)\nabla_{\theta }Q\left(s,a;\theta \right). \end{array}$$

Here, target_DQN_ is a target value computed by

$$\begin{array}{l} \text{targe}{\text{t}}_{\text{DQN}}=r\left(s,a,s^{\prime }\right)+\gamma \underset{a^{\prime }}{\;\max}T\left(s^{\prime },a^{\prime }\right), \end{array}$$

where *T*(*s, a*) represents the target network. Three alternatives can be chosen to update the target network. The first one is periodic update, i.e., the target network is synchronized with the current Q function at every *C* learning step when the following condition is satisfied:

$$\begin{array}{l} \text{total}\_\text{steps}\;\mathrm{mod}\;C\;=0,\;T\leftarrow Q, \end{array}$$

where total_steps represents the total number of updates applied to the Q function up to the present time. This method is based on Neural Fitted Q Iteration (Riedmiller, 2005), which is a batch Q learning method that employs a neural network. During the interval from the previous update to the next update of the target network, the learning is supervised wherein the target is given by the immutable network *T*. The second alternative is symmetric update, in which the target network is updated symmetrically as the learning network, and this is introduced in double Q learning (van Hasselt, 2010; van Hasselt et al., 2016). The third possible choice is Polyak averaging update, where the parameter of the target network is updated by the weighted average over the past parameters of the learning network, and this was used, for example, in Lillicrap et al. (2016). In our experiments, we examined DQN using a periodic update of the target network.

In addition to using the target network, DQN utilizes the previously proposed Experience Replay (ER) (Lin, 1993), which is a heuristic that temporarily stores to memory a record of state transitions during a certain number of steps and randomly selects a data point from memory for learning so that the correlations between samples are reduced and sample efficiency is increased through the reuse of data. Specifically, when the agent selects an action *a* at a state *s* and receives a reward *r* and the state then transits to *s*′, this data point (*s, a, r, s*′) is stored in replay memory *D* and used for mini-batch learning. In mini-batch learning, the parameters are updated based on a certain number of data points randomly selected from the replay memory *D*, and this procedure is repeated several times. This makes it possible to prevent stagnation of learning as a result of correlation between data points while maintaining the one-step Markov property. Therefore, the update rule of DQN with ER is given by

(3) $$\begin{array}{r} \theta \leftarrow \theta +\alpha {𝔼}_{\left(s,a,r,s^{\prime }\right)\sim U\left(D\right)}\left[\left(\text{targe}{\text{t}}_{\text{DQN}}-Q\left(s,a;\theta \right)\right)\nabla_{\theta }Q\left(s,a;\theta \right)\right], \end{array}$$

where (*s, a, r, s*′) ~ *U*(*D*) indicates that an experienced sample (*s, a, r, s*′) is drawn uniformly at random from the replay buffer *D*. The learning process of DQN is more stable than that of Q learning because the update rule of DQN introduces a delay between the time when *Q*(*s, a*; θ) is updated and the time when *T*(*s, a*) is updated. Although the use of the target network is critical for stable learning, it hinders fast learning due to this delay.

### 3.2. Techniques Together With DQN

After the DQN work, there have been additional modifications and extensions such to enhance the speed or stability of DQN. One is the dueling network architecture (Wang et al., 2016) that has a neural network architecture with two parts to produce separate estimates of state-value function *V*(*s*) and advantage function *A*(*s, a*). More specifically, the action-value function is decomposed as

(4) $$\begin{array}{r} Q\left(s,a;\theta \right)=V\left(s;\theta_{V}\right)+\left(A\left(s,a;\theta_{A}\right)-\frac{1}{\left|A\right|}\underset{a^{\prime }}{\sum }A\left(s,a;\theta_{A}\right)\right), \end{array}$$

where θ_*V*_ and θ_*A*_ are respectively, the parameters of the state-value function and of the advantage function, and θ = {θ_*v*_, θ_*A*_}. It is experimentally shown that the dueling network architecture converges faster than the conventional single-stream network architecture (Wang et al., 2016; Tsurumine et al., 2019).

Another important technological advance is entropy-based regularization (Ziebart et al., 2008; Mnih et al., 2016) that has been shown to improve both exploration and robustness, by adding the entropy of policy to the reward function. The role of the entropy is to discourage premature convergence and encourage policies to put probability mass on all actions. Soft Q-learning (Haarnoja et al., 2017) is one of off-policy algorithm that maximizes the entropy regularized expected reward objective, and its update rule is given by

(5) $$\begin{array}{r} \theta \leftarrow \theta +\alpha {𝔼}_{\left(s,a,r,s^{\prime }\right)\sim U\left(D\right)}\left[\left(\text{targe}{\text{t}}_{\text{SQL}}-Q\left(s,a;\theta \right)\right)\nabla_{\theta }Q\left(s,a;\theta \right)\right], \end{array}$$

where target_SQL_ is the target value of Soft Q-learning computed by

(6) $$\begin{array}{r} \text{targe}{\text{t}}_{\text{SQL}}=r\left(s,a,s^{\prime }\right)+\frac{\gamma }{\beta }\ln\;\underset{a^{\prime }}{\sum }\exp\left(\beta T\left(s^{\prime },a^{\prime }\right)\right). \end{array}$$

Here, β is a predefined hyperparameter. Note that ln ∑exp() is a smoothened alternative to the maximum function, and the target value of Soft Q-learning (6) converges to that of DQN (3) as β → ∞.

### 3.3. Temporal Consistency Loss

Pohlen et al. (2018) pointed out that DQN is still unstable as the discount factor γ approaches 1 because the temporal difference between non-rewarding subsequent states tends to be ~0. This also makes the learning process unstable, especially in long-horizon MDPs, because unnecessary generalization happens between temporally adjacent target values. Fujimoto et al. (2018) pointed out that the variance of the target value can grow rapidly with each update when γ is large. To resolve this instability issue, Pohlen et al. (2018) added the following auxiliary loss function called Temporal Consistency (TC) loss:

(7) $$\begin{array}{r} L_{\text{TC}}\left(s^{\prime },{ã}^{*},\theta \right)=\frac{1}{2}{\left(T\left(s^{\prime },{ã}^{*}\right)-Q\left(s^{\prime },{ã}^{*};\theta \right)\right)}^{2}, \end{array}$$

where ${ã}^{*}=\text{argma}{\text{x}}_{a^{\prime }}T\left(s^{\prime },a^{\prime }\right)$. Although Huber loss was adopted in the original paper, L2 loss is used in this paper to make a fair comparison between different methods. The update rule of DQN with TC-loss is given by

$$\begin{array}{l} \theta \leftarrow \theta +\alpha {𝔼}_{\left(s,a,r,s^{\prime })\sim U(D\right)}[\left({\text{target}}_{\text{DQN}}-Q(s,a;\theta )\right)\nabla_{\theta }Q(s,a;\theta )-\text{λ}\nabla_{\theta }L_{\text{TC}}(\theta )], \end{array}$$

where λ is a predefined positive hyperparameter. Note that Pohlen et al. (2018) used TC-loss together with the transformed Bellman operator, which reduces the target's scale and variance, instead of clipping the reward distribution to the interval [−1, 1]; however, we do not adopt the transformed Bellman operator because the hyperparameters of the learning algorithm should be tuned individually for each task.

## 4. Constrained DQN

DQN uses a target network to calculate the target required for updating the Q function. The target network *T* is synchronized with the Q function *Q* at every learning step. Although this heuristic successfully stabilized the learning process, it was often time-consuming for learning because the value was not propagated unless the target network was updated.

In this study, we present Constrained DQN, which not only calculates the target with the current *Q*, as in the case of conventional Q learning, but also constrains parameter updates based on the distance between the current *Q* and the target network as a way to stabilize the learning. When the difference between the outputs of the Q function and the target network is large, Constrained DQN updates its parameters conservatively, as in the case of DQN, but when the difference between the outputs is small, it updates the parameters aggressively, as in the case of conventional Q learning.

In Constrained DQN, the following loss function, which is similar to TC-loss, is considered:

(8) $$\begin{array}{r} L_{\text{CDQN}}\left(s^{\prime },a^{*},\theta \right)=\frac{1}{2}{\left(T\left(s^{\prime },a^{*}\right)-Q\left(s^{\prime },a^{*};\theta \right)\right)}^{2}, \end{array}$$

where $a^{*}=\text{argma}{\text{x}}_{a^{\prime }}Q\left(s^{\prime },a^{\prime }\right)$. The difference between Equations (7) and (8) is that our loss function considers the maximum value of the learning network while TC-loss uses that of the target network. Constrained DQN updates the parameters by the standard Q learning algorithm (2) when the following constraint is satisfied:

(9) $$\begin{array}{r} {𝔼}_{\left(s,a,r,s^{\prime }\right)\sim U\left(D\right)}\left[L_{\text{CDQN}}\left(s^{\prime },a^{*},\theta \right)\right]\leq \eta , \end{array}$$

where η is a positive threshold of the constraint. Otherwise, the loss function (8) is minimized. Consequently, Constrained DQN updates the parameter by

(10) $$\begin{array}{l} \theta \leftarrow \theta +\alpha {𝔼}_{\left(s,a,r,s^{\prime })\sim U(D\right)}[\left({\text{target}}_{Q}-Q(s,a;\theta )\right)\nabla_{\theta }Q(s,a;\theta )-\lambda l(s^{\prime },a^{*};\theta ,\eta )], \end{array}$$

where *l*(*s*′, a^*^; θ, η) is the gradient of the regularization term, defined by

$$\begin{array}{l} l\left(s^{\prime },a^{*};\theta ,\eta \right)=\{\begin{array}{cc} 0 & \text{if }{𝔼}_{\left(s,a,r,s^{\prime }\right)\sim U\left(D\right)}\left[L_{\text{CDQN}}\left(s^{\prime },a^{*},\theta \right)\right]\leq \eta \\ \nabla_{\theta }L_{\text{CDQN}} & \text{otherwise}. \end{array} \end{array}$$

If the constraint condition is satisfied, then *l*(*s*′, a^*^; θ, η) = 0 and the update rule of Constrained DQN is identical to that of Q-learning with experience replay. Similar to DQN with periodic update, the target network is synchronized with the current Q function at every *C* learning step.

Constrained DQN can be used together with the techniques described before. When the target value is computed by

(11) $$\begin{array}{r} \text{targe}{\text{t}}_{\text{CSQL}}=r\left(s,a,s^{\prime }\right)+\frac{\gamma }{\beta }\ln\;\underset{a^{\prime }}{\sum }\exp\left(\beta Q\left(s^{\prime },a^{\prime };\theta \right)\right), \end{array}$$

the update rule is interpreted as Constrained Soft Q learning, which can also be seen as Soft Q learning with the inequality constraint (9).

Table 1 gives the frequency of constraint activation for each learning phase and hyperparameter. As learning progresses and approaches convergence, the difference between the Q function and the target network tends to be small, and so the frequency of activating the constraint decreases and the update rule approaches that of ordinary Q learning. As the value of λ increases, the influence of the constraint term increases, and so the update rule becomes conservative as in the case of DQN. As the value of η increases, it becomes more difficult to activate the constraint, and so the update rule approaches that of ordinary Q learning.

**Table 1:** Frequency of constraint activation for each learning phase and hyperparameter.

**Learning phase**	**Initial phase**	**Final phase**
λ	Big	Small
η	Small	Big
Frequency of activating constraint	High	Low
(update rule)	(like DQN)	(Q learning)

## 5. Experiments Results

In this section, we compare the proposed method and the conventional method using an MNIST maze task, a Mountain-Car task based on OpenAI Gym (Brockman et al., 2016), a robot navigation task, and two Atari games. The state spaces of the MNIST maze, the Mountain-Car, and the robot navigation are a grayscale image, a two-dimensional continuous real-valued vector, and a concatenation of an RGB image and a 360-dimensional LIDAR vector, respectively. The state space of the Atari games is explained later.

### 5.1. MNIST Maze

#### 5.1.1. Task Setting

**Figure 1:**
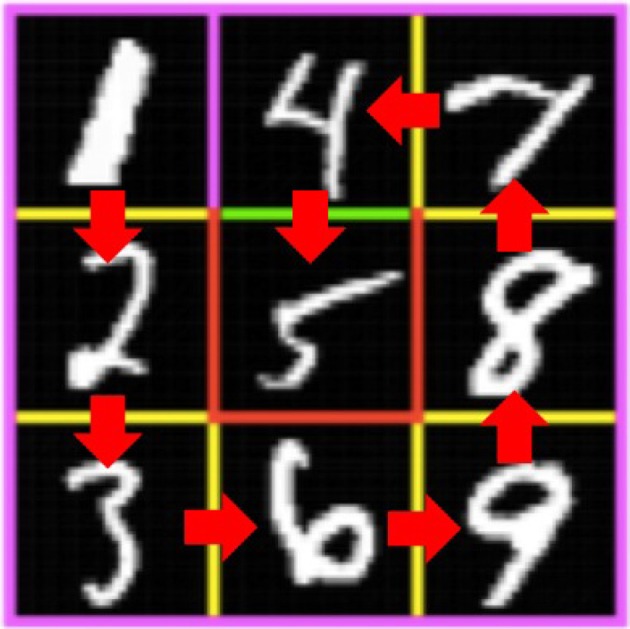
MNIST maze task. The agent aims to reach goal “5” on the 3 × 3 maze by selecting either an up, down, left, or right movement. The lines separating the squares are yellow, green, red, or pink, and they do not change over the episodes. It is impossible to pass through a pink wall, and if the agent selects the direction to a pink wall, the movement is canceled and that agent's position does not change. If the agent reaches “5” across the green line, a + 1 reward is provided, and if the agent reaches “5” across the red line, a − 1 reward is provided. The agent can observe an image of 24 × 24 pixels in which it resides. The number assignment is fixed for all episodes, but the image for each number is changed at the onset of each episode. For example, the upper left tile is always a “1,” but the image of “1” is randomly selected from the training data set of MNIST handwritten digits at the onset of each episode.

The MNIST maze is a 3 × 3 maze (Elfwing et al., 2016) tiled with handwritten number images taken from the MNIST dataset shown in Figure 1. The images in a maze are randomly taken from MNIST for each episode, but the number on each maze square is fixed for all learning episodes. The initial position of the agent is “1” (upper-left square of the maze). In each step, the agent observes the image in which it resides and then chooses an action of up, down, left, or right based on its observation according to the behavioral policy. The agent then moves in its chosen direction in a deterministic manner; however, it is impossible to pass through pink walls, so if the agent selects the direction of going through a pink wall, the movement is ignored and the agent position does not change. If the agent reaches “5” across the green line without going through any red line, a + 1 reward is given, and if the agent reaches “5” across a red line, a − 1 reward is given. The episode ends when the agent reaches “5” or when the agent has performed 1, 000 behavioral steps without reaching “5.” In the latter case, a reward of 0 is given. This task requires both MNIST handwritten character recognition and maze search based on reinforcement learning.

**Figure 2:**
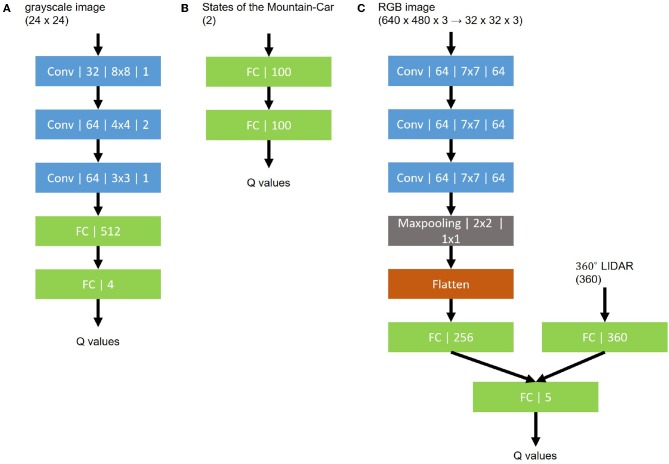
Network structures for the Q function. **(A)** MNIST maze task. **(B)** Mountain-Car task. **(C)** robot navigation task. Every convolutional layer is represented by its type, channel size, kernel size, and stride size. Other layers are represented by their types and dimensions.

We applied Q learning, DQN, DQN with TC-loss, and Constrained DQN to this task to make a comparison. We used a network architecture consisting of the three convolutional layers and two fully connected layers shown in Figure 2A. The input dimensionality is the same as that of the MNIST images, and the output dimensionality was four, which indicates the number of possible actions in this task. According to the ϵ greedy method, ϵ was reduced in the first 1, 000 steps. We set the other parameters, such as the size of replay memory and the parameters of the optimizer, RMSProp [more specifically, RMSPropGraves (Graves, 2013) described in Appendix], to those used in DQN (Mnih et al., 2015). As for the hyperparameters, λ was set to either 0.5, 1, 2, or 5, and η was set to either 10^−2^, 10^−3^, 10^−4^, 10^−5^, 10^−6^, or 0. Training takes about 4 h on a single NVIDIA Tesla K40 GPU for each setting.

#### 5.1.2. Results

**Figure 3:**
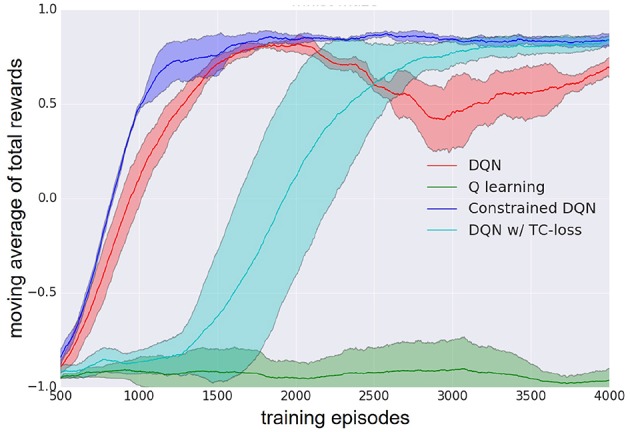
Learning curve of DQN (red), Q learning (green), DQN with TC-loss (cyan), and Constrained DQN (blue) on the MNIST maze task. Here, Q learning refers to DQN without the use of experience reply or the target network. Horizontal axis denotes the number of learning episodes. Vertical axis denotes the moving average of the total reward received in each learning episode. Lightly colored zone represents the standard deviation.

Figure 3 presents the learning curves of Q learning, DQN, DQN with TC-loss, and Constrained DQN. The best hyperparameters of Constrained DQN are λ = 2, η = 10^−5^, and *C* = 10, those of DQN with TC-loss are λ = 1 and *C* = 10, 000, and that of DQN is *C* = 10, 000. We found that the number of samples required for convergence is smaller in Constrained DQN than in the baselines. We also found that DQN had an unstable learning curve after about 2,000 training episodes. DQN with TC-loss yielded a stable learning curve, but it learned much more slowly than did DQN or Constrained DQN. We did not find Q learning to work in the MNIST maze task. Note here that we consistently used the same network architecture, including CNN, even in the case of Q learning.

**Figure 4:**
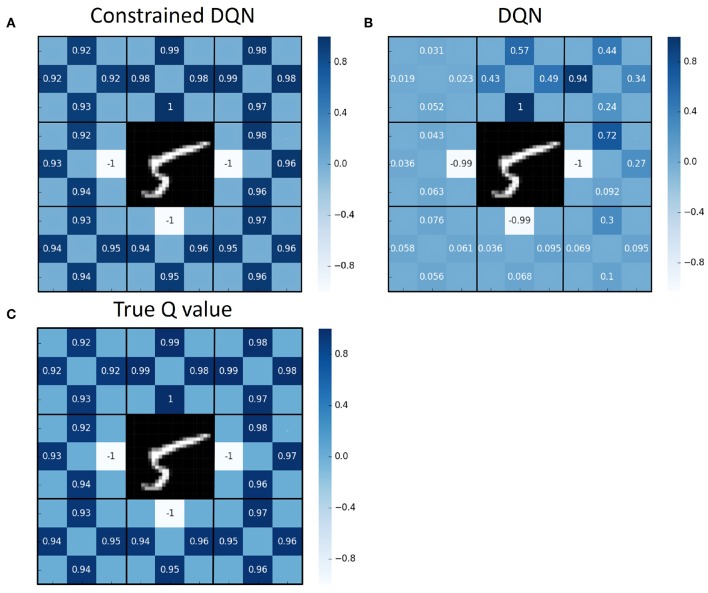
Comparison of values of the Q function for each state and action pair on the MNIST maze task. **(A)** Average Q value obtained by Constrained DQN after 50,000 training *steps* (not training *episodes*). **(B)** Average Q value obtained by DQN. **(C)** True Q value. The values of up, down, left, and right at each state are filled at the corresponding sides of the number position of Figure 1.

**Figure 5:**
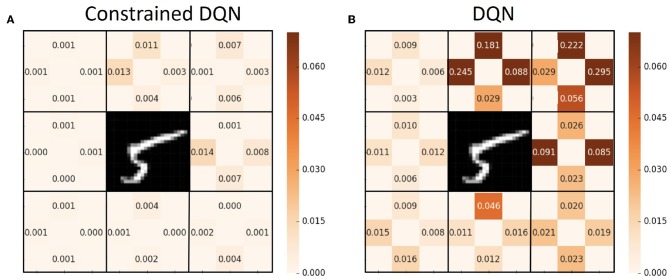
Comparison of variance of the Q function for each state and action pair on the MNIST maze task. **(A)** Variance of Q value obtained by Constrained DQN after 50,000 training steps. **(B)** Variance of Q value obtained by DQN. The values of up, down, left, and right at each state are filled at the corresponding sides of the number position of Figure 1.

Figure 4 presents the average Q value after 50,000 learning steps and the true Q value for each state and action pair. We performed 100 episodes for evaluation, and the Q value averaged over those episodes is shown for each state and action pair. Here, we obtained the true Q values through complete dynamic programming. Figure 5 illustrates the variance of the Q value after 50,000 learning steps. These figures show that the average Q value estimated by Constrained DQN is almost the same as the true Q value, and the variance of the Q value estimated by Constrained DQN is smaller than that of DQN. Although the learned policy obtained by DQN was optimal, the estimated Q function was far from the true one.

**Figure 6:**
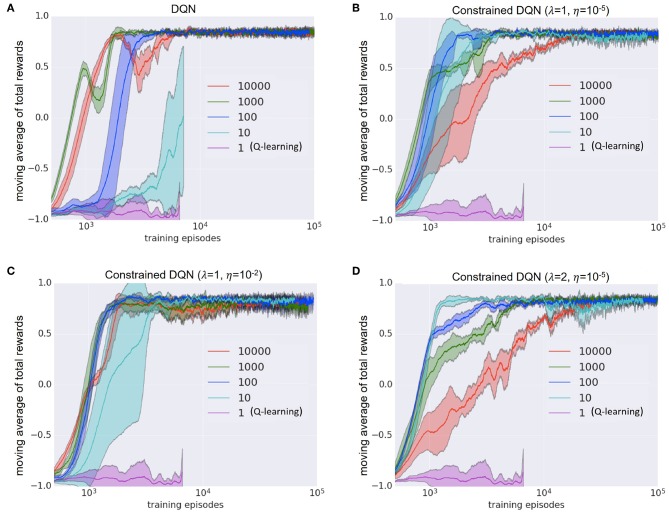
Effects of the update frequency of the target network on the MNIST maze task. **(A)** Learning curves of DQN. **(B)** Learning curves of Constrained DQN with λ = 1 and η = 10^−5^. **(C)** That of Constrained DQN with λ = 1 and η = 10^−2^. **(D)** That of Constrained DQN with λ = 2 and η = 10^−5^. Horizontal axis denotes the number of learning episodes in the logarithmic scale. Vertical axis denotes the moving average of the reward received in each learning episode. The legend indicates the update frequency of the target network. Shaded area represents the standard deviation. Each experiment was performed for 1,000,000 learning steps, and the results of up to 100,000 episodes are displayed.

Figure 6 presents the results when we changed the update frequency of the target network. We examined DQN and our Constrained DQN with three hyperparameter settings. DQN did not converge when the update frequency of the target network was one learning step (that is, Q learning) or 10 learning steps, but Constrained DQN converged regardless of the setting of λ and η when the update of the target network was performed every 10 learning steps. Since the Q learning did not progress well, the average number of steps per episode was large; this is the reason why the number of episodes was <10,000 even in the later part of the 1,000,000 steps. From this result, we consider Constrained DQN to be more robust to changes in the update frequency of the target network than DQN.

**Figure 7:**
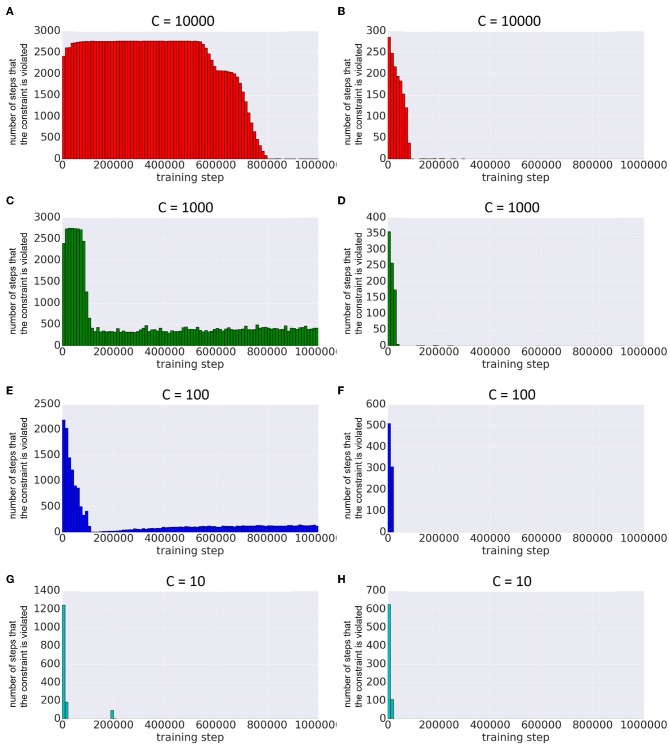
Comparison of the number of steps by which the constraint is violated with different frequencies of updating the target network on the MNIST maze task. Horizontal axis represents the number of learning steps. Vertical axis represents the number of steps in which the constraints were activated within a fixed number of steps. The left column is the result when η = 10^−5^, and the right column is when η = 10^−2^ (λ = 1 in both columns). **(A)** The case of C = 10000 and η = 10^−5^, **(B)** that of C = 10000 and η = 10^−2^, **(C)** that of C = 1000 and η = 10^−5^, **(D)** that of C = 1000 and η = 10^−2^, **(E)** that of C = 100 and η = 10^−5^, **(F)** that of C = 100 and η = 10^−2^, **(G)** that of C = 10 and η = 10^−5^, and **(H)** that of C = 10 and η = 10^−5^.

Figure 7 shows the number of times the constraint was activated along the learning steps by Constrained DQN. The figure presents the results for two different settings of hyperparameters η. In both cases, the constraint was activated many times in the early stages of learning, but the number of activations decreased as learning progressed. From this result, it is clear that the constraint was effective in the early learning stages, and the learning was equivalent to the unbiased Q learning in later stages. When η is large, it is easy to satisfy the constraint: the smaller the η, the more the constraint was activated.

**Figure 8:**
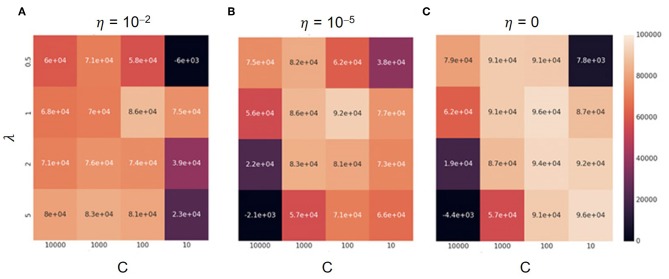
Total rewards across different parameter settings on the MNIST maze task. Darker colors depict low total rewards and lighter colors depict higher ones. In each panel, the horizontal and vertical axes denote the update frequency of the target network and the λ value, respectively. **(A)** The case of η = 10^−2^, **(B)** that of η = 10^−5^, and **(C)** that of η = 0.

Figure 8 is a heatmap of the sum of rewards received throughout learning for each combination of λ, η, and the update frequency of the target network in Constrained DQN. When the update frequency of the target network was large, the square distance between the Q function and the target network was likely large. In this case, the constraint was frequently activated so that the convergence was delayed, especially when the threshold value η was small. When the update frequency was small, on the other hand, the square distance hardly increased, especially when η was large. In such a case, the update method was close to that of conventional Q learning even in the early stages of learning, which made learning unstable. However, when λ = 1, the results were reasonably good regardless of the hyperparameter setting.

### 5.2. Mountain-Car

#### 5.2.1. Task Setting

Mountain-Car is a classical control task, and it has often been used for evaluating reinforcement learning algorithms. The agent (i.e., the car) aims to reach the fixed goal *x* = 0.5 (the top of the hill) from the fixed start position *x* = −0.5 (at almost the bottom of the valley). The agent can observe its current position and velocity. Position is limited to the range [−1.2, 0.6], velocity is limited to the range [−0.07, 0.07], and time is discretized. Available actions at each discretized time include “left acceleration” (*a* = 0), “no acceleration” (*a* = 1), and “right acceleration” (*a* = 2). After determining the action (*a*), velocity is calculated as follows:

$$\begin{array}{l} v=v+0.001\left(a-1\right)-0.0025\cos3x, \end{array}$$

where *v* is velocity and *x* is position. Even if the agent continues to choose “right acceleration” from the start position, the agent cannot reach the goal because of insufficient motion energy. To reach the goal, the agent must choose “left acceleration” first and accumulate enough potential energy on the left-side slope, which is then transformed into motion energy to climb up the right-side hill. The agent is given a − 1 reward at every discretized time step. If the agent reaches the goal, the episode ends; that is, the problem is defined as a stochastic shortest path problem.

We applied Q learning, DQN, DQN with TC-loss, and Constrained DQN to this task and made a comparison. We used a network architecture consisting of the two fully connected layers shown in Figure 2B. The input dimensionality is two, current position and velocity, and the output dimensionality is three, corresponding to the number of possible actions. According to the ϵ greedy method, ϵ was reduced in the first 10, 000 steps and then fixed at a constant value. We set the replay memory size to 10, 000, target network update frequency to 100, λ to 2, and η to 10^−3^. We set the other parameters, such as the RMSProp parameters, except ξ, to those used in DQN (Mnih et al., 2015). It took about 6 h to train each method on a standard multi-core CPU (16-core/32-thread, 2.4 GHz, and 256 GB RAM).

#### 5.2.2. Results

**Figure 9:**
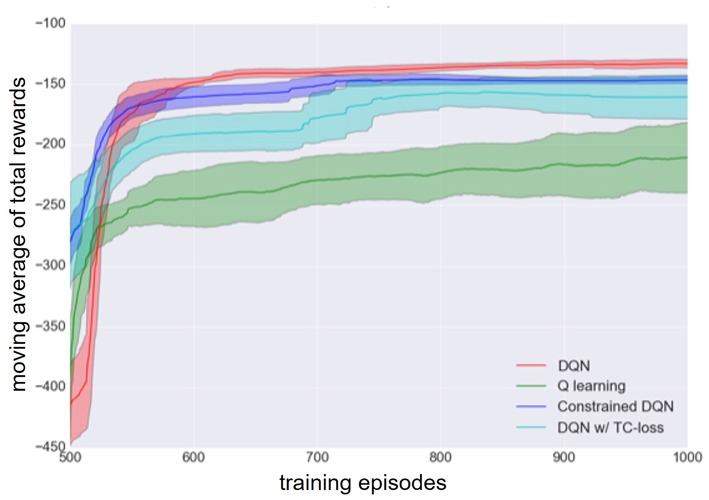
Learning curves of DQN (red), Q learning (green), DQN with TC-loss (cyan), and Constrained DQN (blue) on the Mountain-Car task. Horizontal axis denotes the number of learning episodes. Vertical axis denotes the moving average of the total reward received in each learning episode. The shaded area represents the standard deviation.

**Figure 10:**
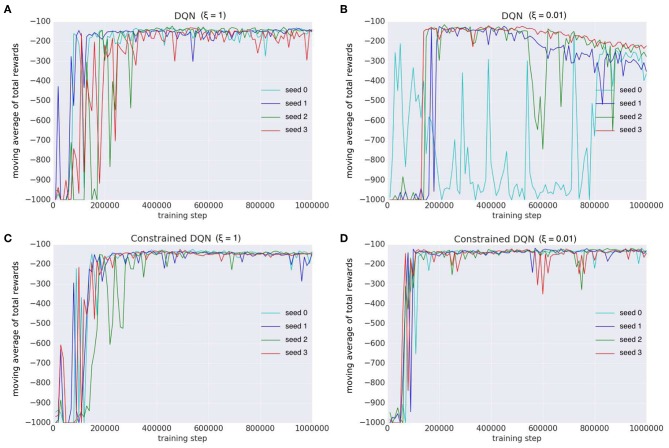
Comparison of learning curves with different random seeds on the Mountain-Car task. Each color indicates the learning curve for one random seed. We examined DQN and our Constrained DQN with two settings of ξ, which is the parameter of the optimizer (RMSProp). Horizontal axis denotes the number of learning *steps* (not learning *episodes*). Vertical axis denotes the moving average of the reward received in each learning episode. **(A)** The learning curves of DQN (ξ = 1), **(B)** those of DQN (ξ = 0.01), **(C)** those of Constrained DQN (ξ = 1), and **(D)** those of Constrained DQN (ξ = 0.01).

Figure 9 shows the learning curves of Q learning, DQN, DQN with TC-loss, and Constrained DQN. We conducted four independent runs for each algorithm. The lines excepting DQN indicate the moving average across four independent runs. One run of DQN was excluded because it failed to learn. We found that Constrained DQN performed better than DQN with TC-loss and Q learning, and learned faster than DQN although its performance was lower than that of DQN. DQN with TC-loss converged faster and achieved more stable learning than Q learning. Although DQN achieved the highest total rewards, its learning process was unstable. Figure 10 shows the learning curves of DQN and Constrained DQN, with two different settings of ξ, which is one of the RMSProp parameters. We observed that the number of samples required for convergence was smaller in Constrained DQN than in DQN for both settings of ξ. We could also observe that when ξ = 0.01, the original DQN was unstable and even degraded after attaining a temporary convergence. On the other hand, our Constrained DQN demonstrated relatively good stability. The learning curves of DQN with TC-loss and Q-learning are shown in Figure S1.

**Figure 11:**
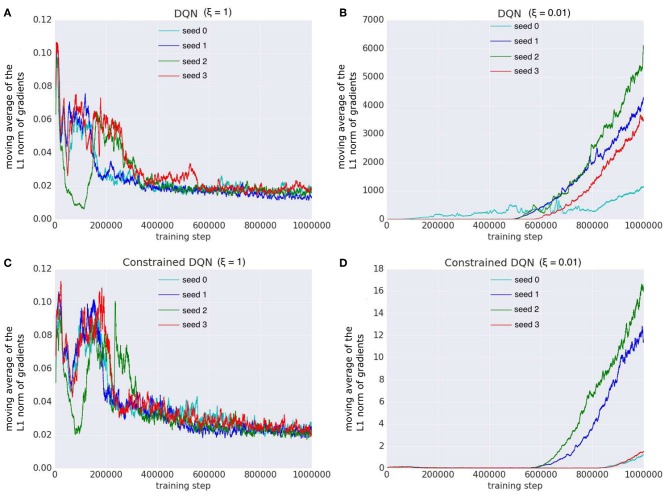
Comparison of the L1-norm of gradients of the last fully connected layer with different random seeds on the Mountain-Car task. We examined DQN and our Constrained DQN with two different settings of ξ, which is the parameter of the optimizer (RMSProp). Horizontal axis denotes the number of learning steps, and vertical axis denotes the moving average of the L1-norm of gradients for the last fully connected layer. **(A)** The L1-norm gradients of DQN (ξ = 1), **(B)** those of DQN (ξ = 0.01), **(C)** those of Constrained DQN (ξ = 1), and **(D)** those of Constrained DQN (ξ = 0.01).

Figure 11 shows how the L1-norms of gradients of the last fully-connected layer changed during learning. When ξ = 1, in the original DQN and Constrained DQN, the number of parameter updates was small. When ξ = 0.01, on the other hand, the number of updates by the original DQN was much larger than that of Constrained DQN. We consider that this is the cause of instability in the original DQN. Because the target value was fixed for *C* learning steps in the original DQN, the variance of gradient decreased rapidly within the *C* steps, making it very small, especially at the end of the *C* steps. On the other hand, because the target value changed every step in Constrained DQN, the variance of gradient smoothly became small through the entire learning process. ξ worked only when the variance of gradient was very small. In such situations, the smaller the ξ, the larger was the number of parameter updates. Consequently, it is considered that in the original DQN, ξ worked too frequently and thus the resulting large number of updates made learning unstable. Actually, with a small ξ (ξ = 0.01), the DQN learning was unstable. On the other hand, Constrained DQN was more robust regardless of the setting of ξ because the variance of gradient decreased smoothly throughout the entire learning process.

### 5.3. Robot Navigation Task

#### 5.3.1. Task Setting

**Figure 12:**
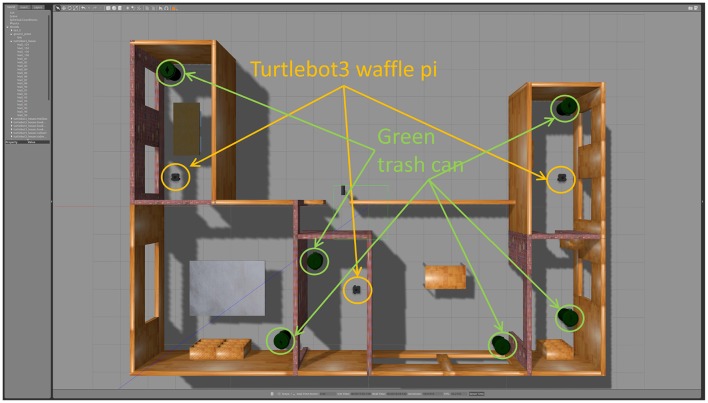
Robot navigation task. Three mobile robots (Turtlebot3 waffle pi), six green trash cans, and various objects were placed in the environment. The objective of the robot is to move to one of the green trash cans without colliding against other objects, including obstacles.

Constrained DQN is evaluated on the robot navigation task shown in Figure 12, in which the environmental model is provided by the task's official site (Robotis e-Manual, 2019). The environment consists of six rooms with six green trash cans, three tables, and three bookshelves. We use a Turtlebot 3 waffle pi platform equipped with a 360° LIDAR. The robot has five possible actions at every step: (1) move forward, (2) turn left, (3) turn right, (4) rotate left, and (5) rotate right. The objective is to navigate to one of the green trash cans placed in the environment. The robot receives a positive reward of +10 for reaching the trash can but a negative reward of −1 for hitting an obstacle. If the robot hits an obstacle, it remains in its current position. If the robot reaches the trash can, the position of the robot is re-initialized randomly in the environment.

We applied Q learning, DQN, DQN with TC-loss, Double DQN (DDQN), Soft Q learning (SQL), and Constrained DQN. The Q function is implemented by the two-stream neural network shown in Figure 2C, in which the input is the values measured by the RGB image and the LIDAR and the output is a five-dimensional vector representing action values. To collect experience efficiently, we use three robots that share the Q function, i.e., one ϵ greedy policy derived from the Q function controls three robots independently and they collect experiences that are sent to the replay buffer. In that sense, this is a simplified implementation of the large-scale distributed optimization that is known to accelerate the value learning (Levine et al., 2016). We conducted five independent runs for each method. Every run included 100 episodes, and each episode had at most 10,000 steps. The memory size of the replay buffer was 30,000 steps. The target network was updated every 3,000 steps. The training batch size was 32, uniformly sampled from the memory buffer. The hyperparameters were α = 0.01, γ = 0.99, ϵ = 0.1, β = 10. We used Ubuntu Linux 16.04 LTS as the operating system and version 375.66 of the NVIDIA proprietary drivers along with CUDA Toolkit 8.0 and cuDNN 6. Training takes about 1 day on a single NVIDIA Tesla P100 GPU for one setting.

#### 5.3.2. Results

**Figure 13:**
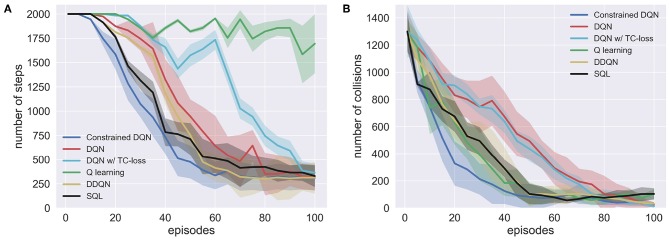
Learning curves of Constrained DQN, DQN, DQN with TC-loss, Q learning, Double DQN (DDQN), and and Soft Q learning (SQL) on the robot navigation task. Here, Q learning refers to DQN without the use of experience reply. **(A)** Number of steps to reach the green trash can. **(B)** Number of collisions with obstacles. Horizontal axis denotes the number of learning episodes. Vertical axes denote, respectively, the number of steps and that of collisions. Shaded area represents the standard deviation.

Since there are two sources of rewards and experience was collected by multiple robots asynchronously, we evaluated the learned Q function every five episodes by executing a greedy policy. Figure 13 shows the number of steps used to go to one of the green trash cans and the number of collisions. This shows that Constrained DQN obtained nearly the fewest steps, the lowest collision rates, and the fastest convergence of learning among the methods compared here. Although there was not much difference in the number of steps between DQN with and without TC-loss, the number of steps for DQN decreased faster than that for DQN with TC-loss. Q learning learned collision avoidance behaviors faster than DQN, but it completely failed to learn about approaching the trash cans. The performance of DDQN at the end of episode is comparable to that of Constrained DQN, but DDQN learned relatively slower than Constrained DQN.

### 5.4. Atari Games

Finally, we investigate whether Constrained DQN can be combined with useful and existing techniques to improve sample efficiency. Here, we examined two techniques. One is the dueling network architecture, and the other is entropy-based regularization.

#### 5.4.1. Task Setting

We evaluate Constrained DQN in two Atari 2600 games, namely Ms. Pac-Man and Seaquest, which were also evaluated by Pohlen et al. (2018). The goal of Ms. Pac-Man is to earn points by eating pellets while avoiding ghosts. Seaquest is an underwater shooter and the goal is to destroy sharks and enemy submarines to rescue divers in the sea. In these experiments, Constrained DQN and DQN with TC-loss were evaluated as the standard algorithm. For each algorithm, the dueling network architecture and the entropy regularization were selected to examine whether Constrained DQN can be used together with such useful techniques developed for improving learning processes. Consequently, we applied six algorithms on the Atari games. Note that TC-loss with the entropy regularization and that with the dueling network architecture are identical to Soft Q learning with TC-loss and Dueling DQN with TC-loss, respectively.

Although the original games are deterministic, randomness was added to introduce uncertainty to the starting state, by performing a random number of no-op actions on initialization. We used the same network architecture used by Mnih et al. (2015) for the standard algorithms and those with the entropy regularization, and the dueling network architecture used by Wang et al. (2016) for the algorithms with the dueling network architecture. The input at each time step was a preprocessed version of the current frame. Preprocessing consisted of gray-scaling, down-sampling by a factor of 2, cropping the game space to an 80 × 80 square and normalizing the values to [0, 1]. We stack four consecutive frames together as a state input to the network and clip the reward to the range of [−1, 1]. We adopted the optimizer and hyperparameters of Pohlen et al. (2018) and β = 1 for the entropy regularization.

#### 5.4.2. Results

**Figure 14:**
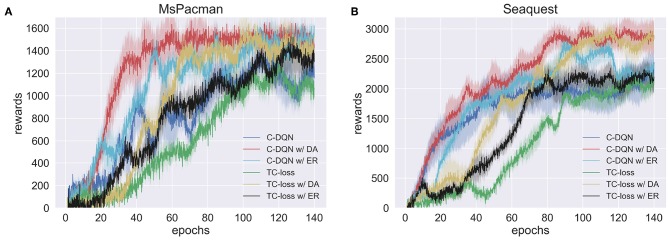
Average performance over ten experiments of the standard Constrained DQN (C-DQN), C-DQN with the dueling architecture (C-DQL w/ DA), C-DQN with the entropy regularization (C-DQL w/ ER), the standard TC-loss, TC-loss and the dueling architecture (TC-loss w/ DA), and TC-loss and the entropy regularization (TC-loss w/ ER). Here, TC-loss refers to DQN with TC-loss. **(A)** Ms. PacMan. **(B)** Seaquest. Shaded area represents one standard deviation from the mean.

Figure 14 shows that Constrained DQN with the dueling network architecture achieved higher rewards faster than other methods on both games. The learning speed of Constrained DQN with entropy regularization was comparable to that of Constrained DQN with the dueling network architecture at the early stage of learning, but its learning process was less stable on Ms. PacMan. However, usage of the entropy regularization did not improve the performance on Seaquest, and it achieved almost the same performance as that of the normal Constrained DQN at the end of learning. This might have occurred, because the hyperparameter of the entropy regularization, β, was fixed during learning.

The experimental results also show that TC-loss and its variants performed worse than the corresponding Constrained DQNs. The performance of TC-loss with the dueling network architecture was comparable to that of Constrained DQN at the end of learning, but it took more epochs to converge.

## 6. Discussion

We performed experiments involving four kinds of tasks, the MNIST maze, Mountain-Car, robot navigation, and two Atari games. Through these experiments, we demonstrated that Constrained DQN converges with fewer samples than does DQN with and without TC-loss and Q learning and, moreover, that Constrained DQN is more robust against changes in the target update frequency and the setting of important parameters of the optimizer, i.e., ξ.

Because the proposed method is a regularization method for DQN, it can more efficiently solve any problem to which DQN has been applied. The experimental results on the Atari games show that Constrained DQN can be used together with the dueling network architecture and the entropy regularization. We think that Constrained DQN can be combined with other techniques that employ a target network, such as improved experience replay techniques (Schaul et al., 2015; Andrychowicz et al., 2017; Karimpanal and Bouffanais, 2018), parametric function of the noise (Fortunato et al., 2018; Plappert et al., 2018), and modified Bellman operators (Bellemare et al., 2016; Pohlen et al., 2018). It suggests that Rainbow Constrained DQN and Ape-X Constrained DQN are respectively considered as an alternative of Rainbow DDQN (Hessel et al., 2017) and Ape-X DQN (Horgan et al., 2018), in which DDQN is replaced with Constrained DQN. Recently, van Hasselt et al. (2019) showed that data-efficient Rainbow outperformed model-based reinforcement learning by extensively using the replay buffer to train and improve the reinforcement learning agent on the Atari games. Their study is impressive because no algorithmic changes were required in its implementation. It is promising to evaluate data-efficient Constrained DQN under the same setting of van Hasselt et al. (2019).

However, it is not trivial to integrate Constrained DQN with DDQN and its extension called Weighted Double Q learning (Zhang et al., 2017), because in these methods the target network was used to decompose the max operation into action selection and action evaluation. To reduce the problem of overestimation, the mellowmax operator (Kim et al., 2019) is promising, which is a variant of Soft Q learning.

Speedy Q learning (Azar et al., 2011) is based on a similar idea to that of our study. Speedy Q learning uses the difference between the output of the current Q function and the previous step's Q function as the constraint. Because the authors of that method only examined the task of discrete state space, one may wonder whether it could be applied to learning with function approximation. In our study, deep/shallow neural networks were used for function approximation so that the results could be verified by tasks of both discrete state space and continuous state space. He et al. (2017) is another method of adding a constraint to Q learning. In this method, the accumulated reward is added to the data saved in the replay memory to allow the upper and lower limits of the Q function to be estimated at each learning step. Averaged DQN (Anschel et al., 2017) is similar to our method because both methods use past Q functions. Averaged DQN uses Q functions of the past few steps for calculating its output, i.e., action values, as the average of the outputs of the past Q functions. This averaging is effective in reducing the variance of the approximation error so that learning can be stabilized. One possible drawback of this method is the necessity of maintaining multiple Q functions, which are often represented as costly neural networks. On the other hand, our method requires only two networks, the current Q function and the target network, as in DQN, and so the number of parameters is not so large.

## 7. Concluding Remarks

In this study, we proposed Constrained DQN, which employs the difference between the outputs of the current Q function and the target network as a constraint. Based on several experiments that include the discrete state-space MNIST maze, the continuous state-space Mountain-Car, simulated robot navigation task, and two Atari games, we showed that Constrained DQN required fewer samples to converge than did the baselines. In addition, the proposed method was more robust against changes in the update frequency of the target network and the setting of important optimizer parameters (i.e., ξ of the RMSProp) than was DQN.

The several tasks used in this study have a discrete action space, but the proposed method can be combined with other methods that are applicable to problems with continuous action space, such as deep deterministic policy gradient (Lillicrap et al., 2016) and normalized advantage function (Gu et al., 2016). If the proposed method could also reduce the number of samples in continuous action space problems, it would be available for a wide range of applications, such as robot control and autonomous driving, since real-world applications involve complications in the collection of a sufficient number of samples for training deep reinforcement learners.

Possible future directions of this study include the following. Although we have shown that our proposed method was sample efficient experimentally, we have not yet established any theoretical reason for Constrained DQN to work properly. Recently, theoretical analyses are made for DQN (Yang et al., 2019) and conservative value iteration (Kozuno et al., 2019). For better understanding of Constrained DQN, we will establish the algorithmic and statistical rates of convergence. In addition, hyperparameter λ was fixed at a heuristic value in this study, but λ could also be optimized under the formulation of the constrained optimization; we can expect an improvement in performance by applying this extension.

## Author Contributions

SO and EU conceived the research. SO, EU, KN, YYas, and SI developed the algorithm. SO and YYam performed the computer simulations. SO, YYam, and EU analyzed the data. SO wrote the draft. SI and EU revised the manuscript. All authors prepared the submitted version.

### Conflict of Interest

SO, EU, KN, and YYas are employed by company Panasonic Co. Ltd, ATR Computational Neuroscience Laboratories, Honda R&D Co. Ltd., and Honda R&D Co. Ltd., respectively. SI was partly employed by ATR Computational Neuroscience Laboratories. These companies are mutually independent commercially or financially. The remaining author declares that the research was conducted in the absence of any commercial or financial relationships that could be construed as a potential conflict of interest.

## Appendix

### Optimizer

In this study, RMSPropGraves (Graves, 2013) is used as an optimizer. When the gradient *f*_*i*_ with respect to θ_*i*_ is given, θ_*i*_ is updated as follows:

$$\begin{array}{l} n_{i}=\rho n_{i}+\left(1-\rho \right)f_{i}^{2},\\ g_{i}=\rho g_{i}+\left(1-\rho \right)f_{i},\\ \Delta \theta_{i}=\beta \Delta \theta_{i}-\frac{\alpha }{\sqrt{n_{i}-{g_{i}}^{2}+\xi }}f_{i},\\ \theta_{i}=\theta_{i}+\Delta \theta_{i}, \end{array}$$

where *n*_*i*_ and *g*_*i*_ are the first- and second-order moments of the gradient and ρ and β give the exponential decay rate. α represents the learning rate, and Δθ_*i*_ is the update amount of θ_*i*_. $n_{i}-{g_{i}}^{2}$ in (1) approximately refers to the moving average of the variance of the gradient. When this term is small (large), the change in parameters is large (small). ξ is a small value that prevents Δ_*i*_ from becoming too large. In all of our experiments, we set α = 0.00025, ρ = 0.95, β = 0.95, and ξ = 0.01, unless otherwise stated.
