# Supplementary material for: Constrained Deep Q-Learning Gradually Approaching Ordinary Q-Learning
Source: Front Neurorobot. 2019 Dec 10;13:103. doi: 10.3389/fnbot.2019.00103 (PMC6914867; doi:10.3389/fnbot.2019.00103)
Supplement: Supplementary file 1 [file Data_Sheet_1.PDF]

Supplementary Material

1 EFFECT OF THE RANDOM SEED IN THE MOUNTAIN CAR TASK

Figure S1 compares learning curves with different random seeds. Constrained DQN and DQN with TC-loss were stable while DQN and Q learning were unstable.

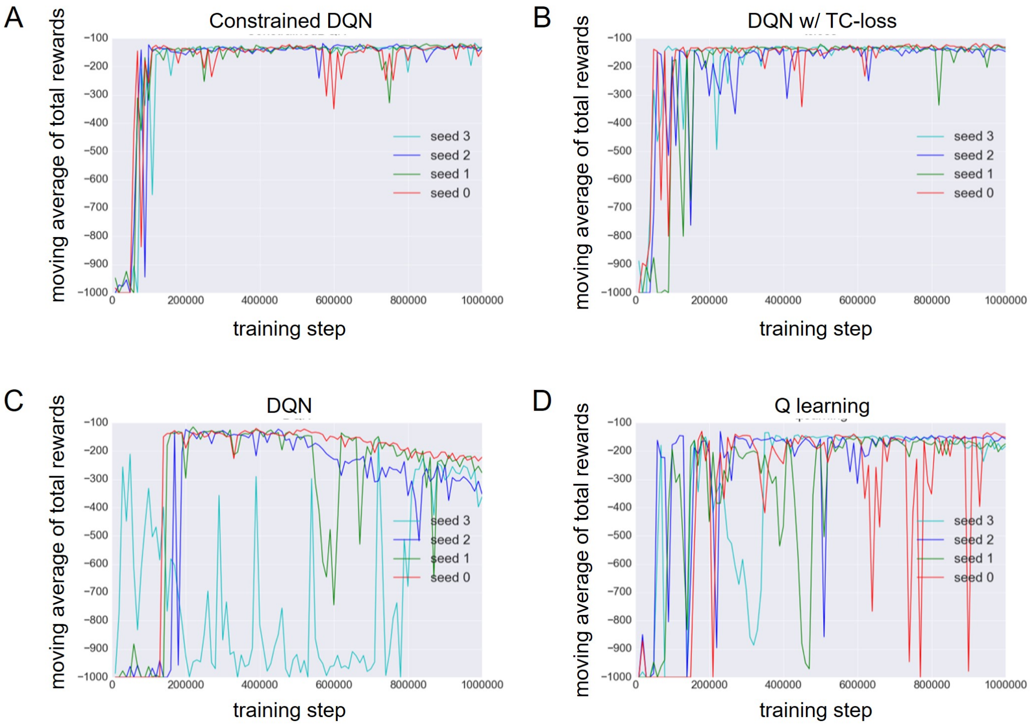

Figure S1. Comparison of learning curves with different random seeds in the Mountain Car task. Each color indicates the learning curve for one random seed. A: Learning curves of Constrained DQN. B: Those of DQN with TC-loss. C: Those of DQN. D: Those of Q-learning. Vertical axis denotes the moving average of the reward received in each learning episode.
